# Synthesis and Properties of Novel Reactive Dyes Comprising Acyl Fluoride Group on Cotton Fabrics

**DOI:** 10.3390/molecules27134147

**Published:** 2022-06-28

**Authors:** Canxing Zhao, Rui Shi, Shouchun Li, Penghui Li, Xiaoxue Zhang, Guolin Tong

**Affiliations:** 1 Jiangsu Co-Innovation Center of Efficient Processing and Utilization of Forest Resources, Nanjing Forestry University, Nanjing 210018, China; canxingzhao@njfu.edu.cn (C.Z.); shirui18362928752@163.com (R.S.); pulp_njfu@126.com (S.L.); liph@njfu.edu.cn (P.L.); zhangxx@njfu.edu.cn (X.Z.); 2 Sino-Italian Research Institute of Nanjing for Chemical Technology Development, Nanjing 210037, China

**Keywords:** acyl fluoride, reactive dyes, cotton fabric, low molecular weight, salt-free

## Abstract

Novel reactive dyes with mono- and bi-acyl fluoride reactive groups have been designed and synthesized, which are obtained by using 2-amino-8-naphthol-6-sulfonic acid or 1-amino-8-naphthol-3,6-disulfonicacid as the coupling component and 4-aminobenzoyl fluoride (PABF) as the diazo component. Their structures have been defined by nuclear magnetic resonance spectroscopy and ultraviolet–visible spectra (UV—Vis). The novel reactive dyes were evaluated on cotton by using the exhaust dyeing method. The properties were examined in detail, and the results showed that the dye concentration of 4% (o.w.f), pH = 9, and salt-free was the most effective condition. The fixation of the novel reactive dyes on cotton was 60.27% and 64.13%, respectively. The micro-fluorine-containing reactive dyes have favorable dyeing properties owing to the covalent bond formed between the reactive group of dyes and the functional group of cotton fibers, which can achieve salt-free dyeing of cotton.

## 1. Introduction

Reactive dye is a kind of water-soluble dye with dual dye matrix and reactive groups, which can establish covalent bonds with hydroxyl groups of the cellulose fabric during the application process [1,2]. Reactive dye has been practically proven to get advantages of bright color, high color fastness, and strong applicability. In 1956, Stephen et al. firstly discovered reactive groups based on cyanuric chloride [3]. Then, Hoechst successfully used vinyl sulfone reactive dyes for dyeing cellulose in 1958 [4]. For vinyl sulfone reactive dyes, the nucleophilic elimination bonus reaction enables the formation of C=C double bonds during the dyeing process, which contributes to good exhaustion and fixation performance [5,6]. However, it cannot be applied under strong alkali conditions. Monochloro triazinyl dye with ring-NHR (or alkoxy) groups was developed in order to replace chloride with triazine for this problem, but the dyeing pH and temperature would need to be increased to solve the competitive hydrolysis problem of reactive dyes. Traditional hydration chemical processes often involve demanding conditions such as strong alkaline solutions and high temperatures (150–160 °C) for baking [7], so most dyes are hydrolyzed in harsh dyeing baths [8,9]. A large amount of stained wastewater hinders the penetration of light leading to the weak photosynthesis of aquatic organisms, and the high content of heavy metals, salt, and organic matter poses great threats to the environment and public [10,11]. Therefore, fluorine-containing reactive dyes with low activity and proper stability have attracted attention.

A big advance was made by Effros L.S and Shouchun Li, who developed sulfonyl fluoride reactive dyes with small reactive groups [12]. Although researchers differ in some details, there is a common understanding that the molecular weight of reactive groups becomes more and more smaller in reactive dyes, which contributes to reducing the proportion of hydrolysis-prone components in reactive dyes and costs. Klanènik [13] found that reactive dyes with monochloro-s-triazine groups are more sensitive than reactive dyes with monofluoro-s-triazine groups by hydrolysis kinetics. Moreover, reactive dyes containing fluoro-substituted triazines have the following advantages compared with those containing chloro-substituted triazines [14,15]: the fixation rate is 10%−15% higher, and the dyeing condition is milder such as lower salt, lower alkali, lower bath ratio, and/or lower energy savings. Due to the strong electron-withdrawing property of the fluorine atom, the carbon atoms connected to the fluorine atom show a stronger positive charge and are more likely to react with fibers. Generally speaking, the reactive dyes containing acyl fluoride groups could enhance the fixation rate and improve the dyeing conditions compared with chlorine-containing reactive dyes [16,17].

In this study, the novel reactive dyes containing reactive groups (—COF) were synthesized through the coupled reaction of 4-aminobenzoyl fluoride with γ acid and H acid. Therein, the amide group could be easily introduced between the color base and the reactive group. The molecular weight of the acyl fluoride group is 47, in stark contrast with those reported with molecular weights beyond 100. As a result, the novel dyes with low molecular weight reactive groups are less reactive but stable in structure as well as low-cost and applicable in mild conditions. Moreover, the tautomerizm (ketone and enamine) could increase the length of the conjugated system (λ_max_). It represents the first trial of micro-fluorine-containing reactive dye by using 4-aminobenzoyl fluoride, which advances the dyeing process with outstanding quality and stability. 

## 2. Results and Discussion

### 2.1. Synthesis and Spectral Properties of the Novel Reactive Dyes

**Scheme 1:**
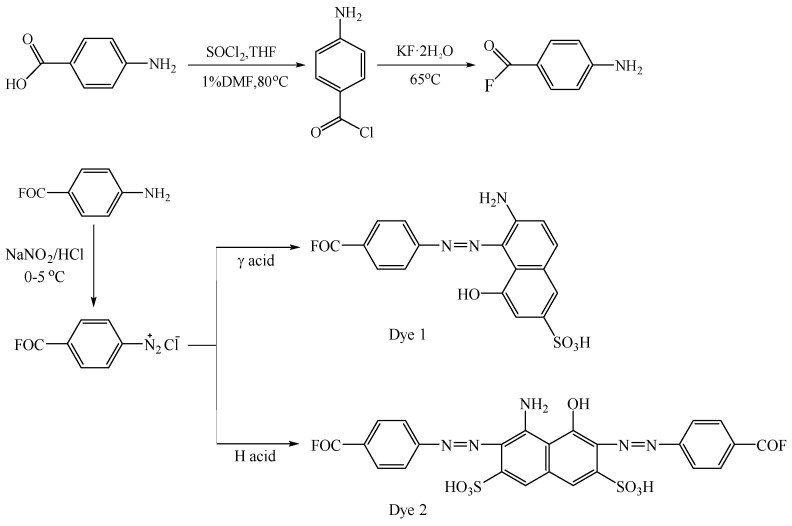
Synthesis routes of the dye intermediate and novel reactive dyes (Dye-1, Dye-2).

In this study, the dye intermediate (PABF, *p*-aminobenzoyl fluoride) and novel reactive dyes (Dye−1, Dye−2) combining azo chromophore and acyl fluoride reactive groups were designed. Meanwhile, the Dye’ containing a carboxyl group was synthesized to compare the difference in dyeing effects between the carboxyl and acyl fluoride groups. The synthesis routes of the dye intermediate and novel reactive dyes are shown in Scheme 1.

#### 2.1.1. Chemical Analysis

The ^1^H NMR, ^13^C NMR, ^19^F NMR spectra of the dye intermediate and the novel reactive dyes were recorded, as shown in the Supplementary Materials. With electron-withdrawing, fluorine is a strong electro-negative group; the adjacent carbon atom is subjected to a strong spin-spin coupling effect which splits the ^13^C NMR signal of acyl fluoride carbonyl into two peaks. The coupling constants, J_C-F_, of the dye intermediate, Dye-1, and Dye-2 were 349, 346, and 423, respectively. The dye intermediate and the novel reactive dyes were analyzed by employing a VERTEX 80V FT–IR spectrometer (Bruker Co., Faellanden, Switzerland) with the traditional KBr pellet sampling method as shown in the Supplementary Materials, respectively (Figures S5, S10 and S15). Since the peak of the acyl halogen ranged from 1720 cm^−1^ to 1815 cm^−1^, the characteristic absorption peak of the acyl fluoride group was at 1797 cm^−1^ in Figure S5. Meanwhile, the characteristic broad peaks appearing around 3400 cm^−1^ indicated the presence of hydroxyl and amino groups in Figures S10 and S15.

#### 2.1.2. Color Characteristics

**Figure 1:**
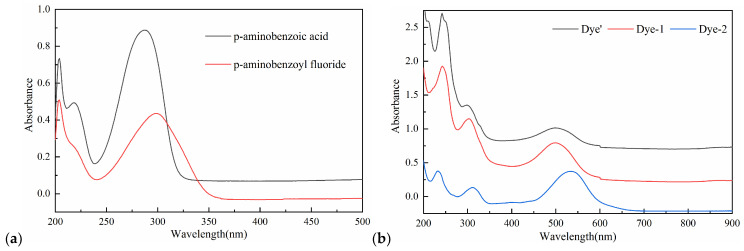
(**a**) UV absorption spectra of PABF and PABA; (**b**) UV absorption spectra of Dye’/Dye-1/Dye-2.

The dye intermediate and the synthesized dyes were prepared into a solution of 0.01 g/L in a deionized water solvent, and the absorption spectra were recorded using a TU-1900 UV–Vis spectrometer as shown in Figure 1. Compared to PABA, the UV absorption spectrum of PABF was slightly shifted to the long wavelength (Figure 1a). It can be attributed to the increase in the mobility of electron clouds when the —COF electron group was connected to the conjugated system. Due to the strong electron-withdrawing property of the fluorine atom, the acyl fluoride group is more stable than the acyl chloride group and more active than the carboxyl group. The introduction of a dye intermediate containing acyl fluorine not only reduces the hydrolysis of dyes, but also improves their fixation rate. Figure 1b indicated the maximum absorbance at 500 nm for Dye-1 with magenta shade and at 531 nm for Dye-2 with burgundy shade, respectively. The introduction of bis-azo chromophores into Dye-2 showed a bathochromic shift of 31 nm from Dye-1, so the dyes exhibited different color characteristics. Besides, the absorption peak of Dye-2 at 531 nm wavelength was obviously higher than that of Dye-1. It reflected that the solubility and the apparent chromaticity of Dye-2 containing bis-acyl fluoride reactive groups were better than that of Dye-1 with the same amount of dye. 

### 2.2. Effect of pH on Dyeing Properties

**Scheme 2:**
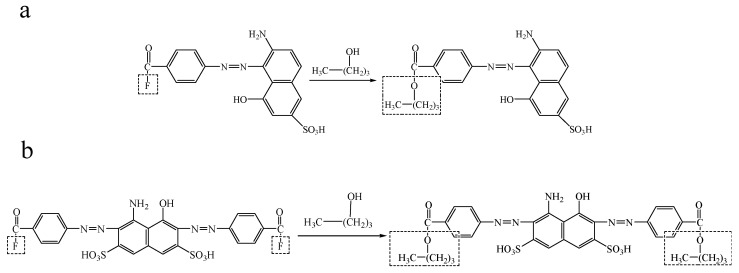
Simulated dyeing process response of Dye-1 (**a**) and Dye-2 (**b**).

The reaction between reactive dyes and cellulose fibers mainly refers to the elimination reaction, hydrolysis reaction, and other side reactions [18]. Zotou et al. [14,19,20,21] studied the hydrolysis kinetics and the alcoholysis kinetics of reactive dyes by high-performance liquid chromatography (HPLC). In addition, alcohols are often used as models for the hydroxyl groups of cellulose during basic research on the chemical selectivity of reactive dyes [22]. As mentioned above, reactive dyes are mainly used for fiber through the reaction of reactive groups with six hydroxyl groups of cellulose fibers to form covalent bonds, so using —OH on *n*-butanol simulates the hydroxyl groups in the fiber that interact with the dye. The reaction process between the two reactive dyes containing acyl fluoride and *n*-butanol is shown in Scheme 2. There was a conjugated system composed of C=C and C=O during the simulated dyeing procedure, which indicated that there was a K absorption band. The large absorption peak was detected with two double bonds in the 252 nm wavelength for Dye-1. At the same time, the large absorption peak with two double bonds was inferred in the 233 nm wavelength for Dye-2. 

**Figure 2:**
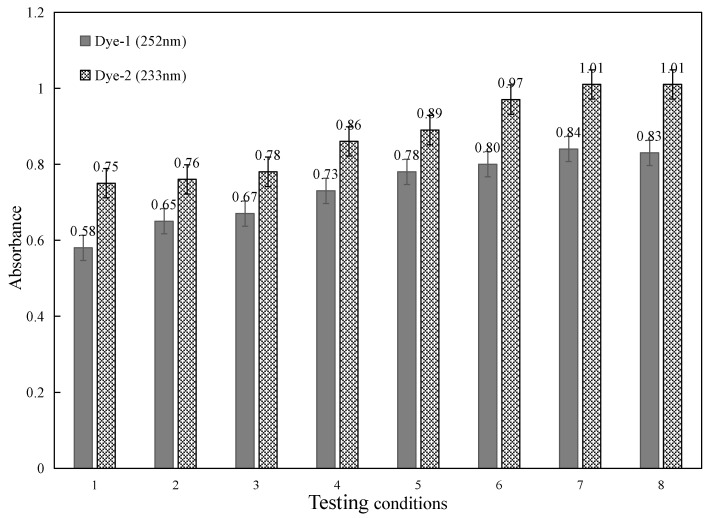
Detection of abs in simulated dyeing experiments. Please note: 1-Mixing of 0.0005 mol dye and 0.0005 mol *n*-butanol at room temperature. 2-Reaction of 30 min with reaction solution 1 at room temperature. 3-Put the 2-reaction solution into a water bath heated to 60 °C and reacted 15 min. Divided the reaction liquid of 3 into 5 parts. The pH was adjusted to 6, 7, 8, 9, 10 with sodium carbonate labeled as 4, 5, 6, 7, 8, then reacted 45 min. The liquid after uniform mixing was taken for the process of UV detection, and the concentration was always same.

The reaction between reactive dyes and cotton knitted fabrics takes place under alkaline conditions. Figure 2 reflects the maximum absorbance value of Dye-1 and Dye-2 with *n*-butanol, which was detected in the 252/233 nm wavelength. As the reaction proceeded, the maximum absorption value of the 252 nm wavelength and the 233 nm wavelength was always increasing. The absorbance of sample 7 was the highest when the reaction solution of Dye-1 or Dye-2 and *n*-butanol was at pH = 9. It showed that the reactivity of Dye-1 and Dye-2 was the best under the condition of pH = 9. At the same time, the absorbance of 4, 5, 6, 7, 8 increased less after a prolonged reaction time of 4, 5, 6, 7, 8 to 90 min or 135 min, so the absorbance did not repeat. In conclusion, the optimized pH value of Dye-1 and Dye-2 was 9 at 60 °C.

### 2.3. Effect of Salts and Time on Dyeing Properties

**Figure 3:**
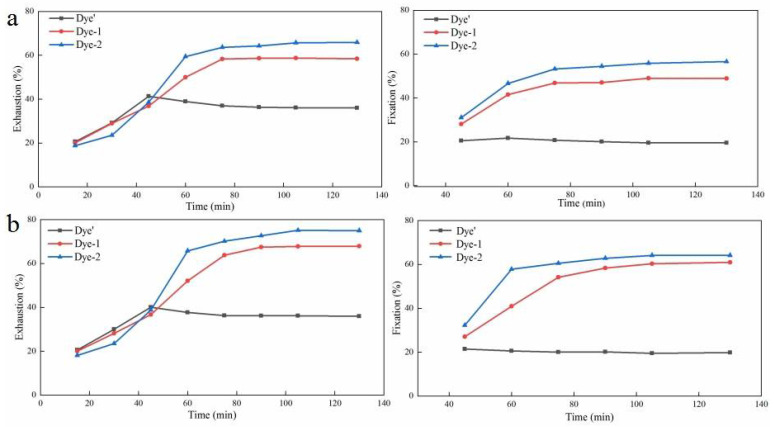
(**a**) Fixation and exhaustion of dyes with added sodium sulfate. (**b**) Fixation and exhaustion of dyes without adding sodium sulfate.

Sodium sulfate, which could adjust the dyeing properties by changing the solubility of dyes and reducing the repulsive force between dyes and fibers, is an important compound for dyeing reactive dyes on cotton knitted fabric [23]. The dyeing time depends on the type of fabric and the required depth and intensity of dyeing. Therefore, the effect of salts and time on dyeing properties is essential to investigate. The results are shown in Figure 3. From Figure 3a, the exhaustion and fixation of the three dyes slightly increased with the increase of time in the range of 15−105 min, while it decreased due to the hydrolysis of the reactive group when the time was more than 105 min. The dye formed a covalent bond with the fiber; extending the fixation time leads to hydrolysis of fixed dyes. The exhaustion and fixation of reactive dyes without sodium sulfate were better, as shown in Figure 3b. Compared with the Dye’, Dye-1 and Dye-2 with acyl fluoride groups were more sensitive to salts and obtained better dyeing properties. Overall, the novel reactive dyes showed less dependence on dyeing time, and low- or no-salt dyeing condition was more suitable.

### 2.4. Dyeing Properties of the Synthesized Dyes

**Scheme 3:**
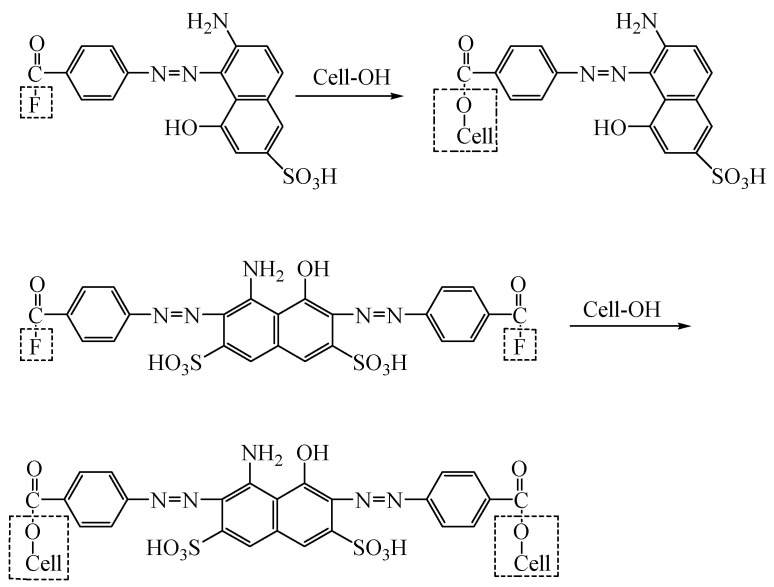
Reaction of the reactive dyes containing acyl fluoride with cellulose.

It is common knowledge that the exhaustion and fixation rate are significant characteristics of dyeing processes. The reactive group in the molecular structure of reactive dyes could form covalent bonds with the hydroxyl groups of cellulose fabric by nucleophilic addition reaction. Therefore, the nature and number of reactive groups have a crucial influence on dyeing properties. The reaction mechanism between the dyes and cellulose is shown in Scheme 3.

At the dye concentration of 4.0%, the color strength (K/S) and characteristic values (SERF) of the synthesized dyes are listed in Table 1. As shown in Table 1, Dye-1 containing the acyl fluoride active group showed higher K/S and fixation efficiency values than that of Dye’, indicating that the acyl fluoride group had better stability than the carboxyl group. Similarly, compared with Dye-1 containing an acyl fluoride group, Dye-2 with bis-acyl fluoride groups showed higher K/S and fixation efficiency values due to the higher fix efficiency. Moreover, the fixation efficiency value of Dye-2 was 64.13%, which was slightly higher than that of Dye-1. At the same time, Dye-1 and Dye-2 showed proper dyeing properties, indicating that the dyes could be combined with fibers under low temperature and weak alkali conditions.

### 2.5. Colour Assessment

**Figure 4:**
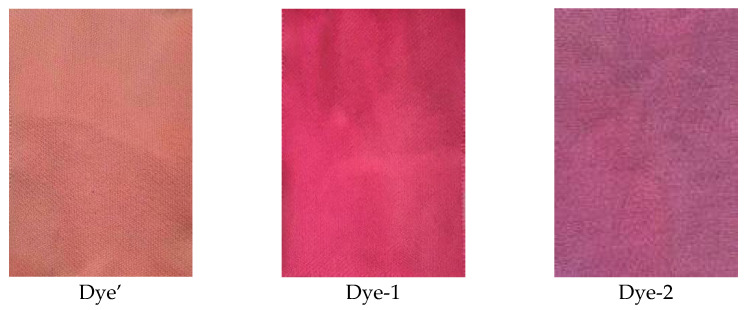
Digital pictures of the cotton knitted fabrics dyed with the synthesized dyes.

As the data show in Table 2, the positive value of the “a*” indicates red of the tone, and the positive and negative value of “b*” indicates yellow and blue of the tone for cotton knitted fabrics. From Table 2, the CIE coordinates of Dye-2 show some blue-green motion as compared to Dye-1. Chroma value denoted as “C*” indicates purity—the lower “C*” value of Dye-2 could be owing to the presence of the bis-chromophoric groups. Similarly, the hue value denoted as “H” is measured in the degree of angle. The values of “H” of the dyed samples showed that the degree of angle is lower than 30° for red color and higher than 270° for blue color in Table 2. Due to the intramolecular combination of two chromophores, the colors of Dye-2 should be the special colors from the mixture color of the blue or the red. The digital pictures of the cotton knitted fabrics dyed with the synthesized dyes are shown in Figure 4.

### 2.6. Fastness Properties

Fastness properties determine the application performance and field of the dyes. Since the dyeing fabric would fade due to washing, rubbing, light, and other factors, it is necessary to measure the fastness properties of dyeing fabric. According to Table 3, the fastness properties of the synthesized dyes on cotton fabric conform to the application requirements. Significantly, the fastness properties of the novel dyes (Dye-1, Dye-2) containing the acyl fluoride groups are better than that of the control dye (Dye’). Moreover, Dye-2 is slightly better than Dye-1 in terms of wash fastness and rub fastness.

## 3. Experimental

### 3.1. Materials and Methods

Sodium nitrite, ammonium chloride, sulfamic acid, anhydrous sodium carbonate, anhydrous sodium sulfate, 1-butanol, thionyl chloride, N, N-dimethyl formamide, and potassium fluoride dihydrate were obtained by *Sinopharm Chemical Reagent Co.* (Shanghai, China). without further purification. Other reagents used for synthesizing the reactive dyes were purchased from Shanghai *Aladdin Biochemical Technology* *Co.* without further purification. All reagents and chemicals used in this work were analytic pure grade. Samples of the cotton knitted fabric used in this study were provided by *Sino Italian Chemitech R&D Institute* (Nanjing, China).

FT–IR spectra analysis was carried out using a VERTEX 80V FT–IR spectrometer (Bruker Co., Faellanden, Switzerland), UV—Vis spectra analysis was performed using a TU-1900 UV—Vis spectrometer (General Instrument Co., Beijing, China). The dye intermediate and novel active dyes were synthesized with further purification and characterized by ^1^H NMR, ^13^C NMR, ^19^F NMR on a Bruker Avance III HD spectrometer 600 MHz by using TMS as internal standard.

### 3.2. Dye Synthesis and Chemical Analysis

**Scheme 4:**
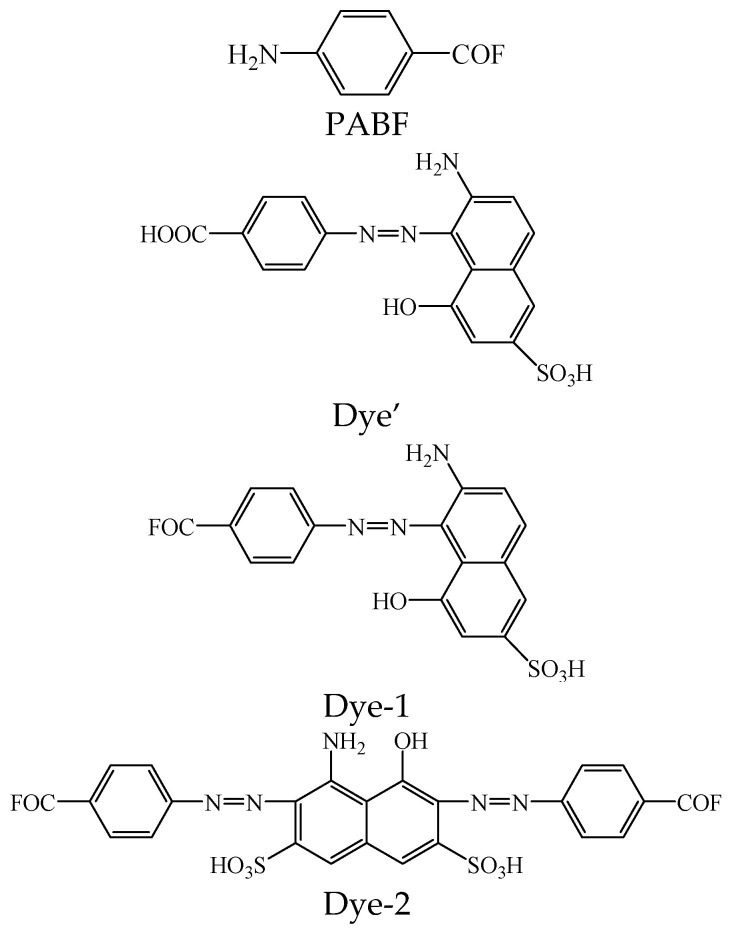
The chemical structures and designations of the dye intermediate and the synthesized dyes.

The chemical structures of the dye intermediate and the novel dyes containing azo chromophores and acyl fluoride groups (Dye’, Dye-1, Dye-2) are shown in Scheme 4.

#### 3.2.1. Synthesis and Analysis of the Dye Intermediate

The dye intermediate was synthesized according to the method described previously [24]. Firstly, 13.72 g purified *p*-aminobenzoic acid (PABA) was poured into a three-necked flask with 30 mL ether, and then 8 mL thionyl chloride was added with stirring. The solution was shaken for 2 h in an ultrasonic generator at 30 °C. Then, the mixture was cooled down to room temperature and distilled by rotatory evaporator. Secondly, the remainder (*p*-aminobenzoyl chloride, PABC) was mixed with 1 mol of KF·2H_2_O at room temperature. The fluoridation was carried out at a temperature of 65 °C for 40 min until the product was completely discolored. Then, the crude product was filtered, washed, dried, and purified by tetrahydrofuran. The structure of the dye intermediate (*p*-aminobenzoyl fluoride, PABF) was characterized by IR and nuclear magnetic spectroscopy.

*p*-aminobenzoyl fluoride (PABF): light yellow solid, yield: 71.51% (4.97 g), m. p.: more than 300 °C. UV: λ_max_ = 287 nm. IR (KBr) (V_max_·cm^−^^1^): 3468 (N—H), 3375 (N—H), 3226 (C—H), 1884 (C—F), 1690 (C=O), 1600 (C=O) cm^−1^. ^13^C NMR (151 MHz, Methanol-d_4_) δ (ppm): 172.20–169.89 (J_C-F_ = 349 Hz), 144.60, 132.02, 127.35, 120.30; ^1^H NMR (600 MHz, Methanol-d_4_) δ (ppm): 2.15 (t, 2H), 7.65–7.68 (m, 2H), 7.95–7.98 (m, 2H); ^19^F NMR (565 MHz, Methanol-d_4_) δ (ppm): 17.52(—COF). Anal. Calcd for C_7_H_6_NOF (139.130): C, 60.43; H, 4.35; N, 10.07; F, 13.66. Found: C, 60.03; H, 4.11; N,10.37; F, 13.78; MS: *m*/*z* 139.

#### 3.2.2. Synthesis and Analysis of the Reactive Dyes

In this study, a well-established protocol from previous studies was utilized for the diazo-coupling reaction [25]. PABF (13.92 g) was mixed with 3.723 g 36.5% hydrochloric acid for diazotization to prepare Dye-1, and the control group named Dye’ was synthesized with PABA. The coupling component containing 0.1 mol of 2-amino-8-naphthol-6-sulfonic acid (γ acid) was added to the solution. The coupling reaction was completed at 5–10 °C for 3 h. For Dye-2, the dosage of PABF and hydrochloric acid was doubled. Due to the particularity of its structure, the first coupling reaction was carried out by adding 0.1 mol 1-amino-8-naphthol-3,6-disulfonic acid (H acid) to the first diazo component solution at 5–10 °C, pH = 1–2 for 2 h. Then, 0.1 mol of the second diazonium solution was added and coupled at 10–20 °C, pH = 5–6. They were based on red γ acid monoazo structure and red H acid disazo structure and named Dye’, Dye-1, and Dye-2. The novel reactive dyes were purified by column chromatography (EtOAc/DCM = 1:20) and characterized by ^13^C NMR, ^1^H NMR, and ^19^F NMR.

Dye-1: red solid, yield: 88.1% (17.135 g), m. p.: 145–150 °C. UV: λ_max_ = 525 nm. ^13^C NMR (151 MHz, DMSO-d_6_) δ (ppm): 167.26–164.97 (J_C-F_ = 345.8 Hz), 129.93, 130.52, 115.41, 153.03, 122.25, 132.61, 120.95, 127.71, 124.88, 115.94, 131.42, 107.43, 146.69, 118.67; ^1^H NMR (600 MHz, DMSO-d_6_) δ (ppm): 8.00–7.98 (d, 2H), 7.45–7.44 (d, 2H), 7.16 (s, 2H), 7.66 (s, H), 8.07(s, H), 7.33 (s, H), 7.56 (s, H), 7.25 (s, H), 7.94 (s, H); ^19^F NMR (565 MHz, DMSO-d_6_) δ (ppm): 18.92. Anal. Calcd for C_17_H_12_N_3_O_5_FS (389.357): C, 52.44; H, 3.11; N, 10.79; F, 4.88; S, 8.23. Found: C, 52.17; H, 3.06; N,10.40; F, 5.18; S, 8.43. MS: *m*/*z* 389.

Dye-2: purple solid, yield: 80.7% (11.763 g), m. p.: 163–167 °C. UV: λ_max_ = 532 nm. ^13^C NMR (151 MHz, DMSO-d_6_) δ (ppm): 167.28–164.48 (J_C-F_ = 422.8 Hz), 130.66, 131.48, 120.97, 154.28, 130.41, 127.73, 115.60, 138.10, 125.09, 131.61, 118.53, 131.95, 117.46, 132.93; ^1^H NMR (600 MHz, DMSO-d_6_) δ (ppm): 8.00 (m, 4H), 7.93 (m, 4H), 6.64 (s, H), 10.40 (s, H), 8.26 (s, 2H), 7.50 (s, H), 7.45 (s, H); ^19^F NMR (565 MHz, DMSO-d_6_) δ (ppm): 20.72. Anal. Calcd for C_24_H_15_N_5_O_9_F_2_S_2_ (619.527): C, 46.53; H, 2.44; N, 11.30; F, 6.13; S, 10.35. Found: C, 46.80; H, 2.69; N, 11.02; F, 5.84; S, 10.43. MS: *m*/*z* 619.

### 3.3. Dyeing Processes and Properties of the Synthesized Dyes

#### Dyeing Procedure

**Figure 5:**
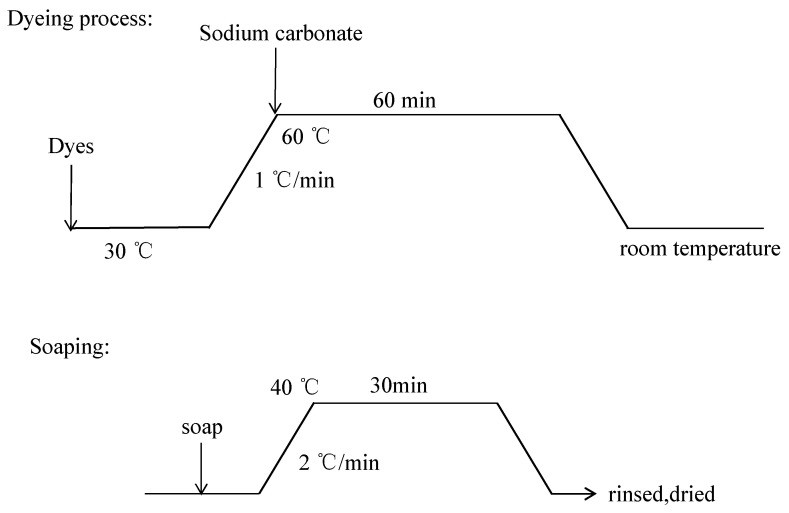
Dyeing experiments of cotton fibers.

All the dyeing procedures were performed on cotton knitted fabrics using the exhaust dyeing method. Dyebath was prepared based on the dye concentration of 4% (o.w.f) and 2 g cotton knitted fabric was immersed into the dyebath at 30 °C with a liquid ratio of 20:1 for 30 min. Then, the dyebath was increased from 30 °C to 60 °C within 15 min, and sodium carbonate was added to regulate it to pH = 9. Finally, the dyed samples were retrieved and rinsed in 5 g/L standard soap solution with the bath ratio of 1:50 at 40 °C. The dyeing procedure and conditions are illustrated in Figure 5.

### 3.4. Test and Analysis of Dyed Cotton Fabric

#### 3.4.1. Color Strength (*K*/*S*) and Characteristic Values (SERF) of Dyed Cotton Fabric

The color strength (*K*/*S* value) of dyed cotton fabrics was calculated using the Kubelka–Munk Equation (1) [26]:

(1) $$K/S=(1-R){}^{2}/2R$$

where *R* is the reflection of the dyed fabric; *K* is the absorption coefficient; and *S* is the scattering coefficient. The reflectance *R* of the dyed fabric was determined on an X-Rite i1 Basic Pro2 spectrophotometer (X-Rite Co., Granville, MI, USA).

The substantivity (*S*%), exhaustion (*E*%), reactivity (*R*%), and fixation (*F*%) of the synthesized dyes on cotton knitted fabric were measured by recording the absorbance of the dye solution at λ_max_ during the dyeing procedure. *S*%, *E*%, *R*%, *F*% were calculated using the following, Equations (2)–(5), respectively.

(2) $$S\%=(A_{0}-A_{1})/A_{0}\times 100$$

(3) $$E\%=(A_{0}-A_{2})/A_{0}\times 100$$

(4) $$R\%=F{}^{\prime }\%/F\%\times 100=(A_{0}-A_{3}-A_{4})/(A_{0}-A_{2}-A_{4})\times 100$$

(5) $$F\%=(A_{0}-A_{2}-A_{4})/A_{0}\times 100$$

where *A*_0_ is the absorbance of the dye solution before dyeing, *A*_1_ is the absorbance of the dye solution after dyeing without fixation, *A*_2_ and *A*_3_ are the absorbances of the dye solution after fixation for 15 min and 60 min, respectively, and *A*_4_ is the absorbance of the washing solution after the soaping step. In addition, *F*′% represents the fixation rate of the dye after fixation for 15 min.

#### 3.4.2. Color Assessment

Color appearance in terms of CIELAB color values were measured by the previous method [27]. Colors are represented by L*, a*, b* coordinates where L* represents lightness, a* represents red-green, and b* represents yellow-blue. The dyed cotton knitted fibers were evaluated on Technidyne Color Touch PC spectral colorimeter.

#### 3.4.3. Fastness Measurement

The washing fastness (ISO 105-C03: 2010), rubbing fastness (ISO 105-X12: 2001), and light fastness (ISO 105-B02: 2013) of the dyed cotton fabrics were examined according to the respective international standards.

## 4. Conclusions

This study provides a preliminary study on reactive dyes containing acyl fluoride groups. Two novel reactive dyes containing azo chromophores and acyl fluoride groups were designed and synthesized. All the dyes were characterized by UV-Vis, ^1^H NMR, ^13^C NMR, and ^19^F NMR. The synthesized dyes, Dye-1 and Dye-2, with magenta and burgundy, respectively, were a combination of different chromophores. In comparison with Dye’ and Dye-1, Dye-2 had a significant advantage in color strength and fastness properties. Moreover, the novel reactive dyes could produce proper dyeing properties under the condition of lower temperature, lower alkali, and salt-free. All these results provide a new approach for the development of fluorine-containing reactive dyes with mild dyeing conditions, less environmental pollution, and favorable dyeing performance.

## Figures and Tables

**Table 1:** Color strength (K/S) and characteristic values (SERF) of the synthesized dyes.

Dyes	K/S	S(%)	E(%)	R(%)	F(%)
Dye’	3.5	30.01	36.11	11.25	19.45
Dye 1	12.5	28.17	67.82	3.71	60.27
Dye 2	14.8	23.46	75.11	3.17	64.13

**Table 2:** Color data and CIE coordinates of the dyes on cotton.

Dye	L*	a*	b*	C*	H	Color
Dye’	14.00	14.45	3.60	14.89	13.99	pink
Dye-1	62.30	29.31	2.63	29.43	5.127	magenta
Dye-2	73.60	11.61	−5.82	12.99	333.4	burgundy

**Table 3:** Washing fastness, rubbing fastness, and light fastness of dyed cotton fabrics.

Dye	Washing Fastness		Rubbing Fastness		Light Fastness
	Change	Stain	Dry	Wet	
Dye’	3–4	3–4	3–4	3–4	4
Dye-1	4	4	4–5	3–4	4–5
Dye-2	4	4	4–5	4	4

## Data Availability

Not applicable.

## Supplementary Materials

The following supporting information can be downloaded at: https://www.mdpi.com/article/10.3390/molecules27134147/s1, Figure S1. Chemical structure of the dye intermediate; Figure S2. ^1^H-NMR spectrum of the dye intermediate; Figure S3. ^13^C-NMR spectrum of the dye intermediate; Figure S4. ^19^F-NMR spectrum of the dye intermediate; Figure S5. IR Spectra of the dye intermediate and 4-aminobenzoic acid; Figure S6. Chemical structure of the Dye-1; Figure S7. ^1^H-NMR spectrum of the Dye-1; Figure S8. ^13^C-NMR spectrum of the Dye-1; Figure S9. ^19^F-NMR spectrum of the Dye-1; Figure S10. IR Spectrum of the Dye-1; Figure S11. Chemical structure the Dye-2; Figure S12. ^1^H-NMR spectrum of the Dye-2; Figure S13. ^13^C-NMR spectrum of the Dye-2; Figure S14. ^19^F-NMR spectrum of the Dye-2; Figure S15. IR Spectrum of the Dye-2.
