# Supplementary material for: Synthesis and Properties of Novel Reactive Dyes Comprising Acyl Fluoride Group on Cotton Fabrics
Source: Molecules. 2022 Jun 28;27(13):4147. doi: 10.3390/molecules27134147 (PMC9268518; doi:10.3390/molecules27134147)
Supplement: Supplementary file 1 [file molecules-27-04147-s001.zip › molecules-1749703-supplementary.pdf]

Supporting Information

Synthesis of novel reactive dyes comprising acyl fluoride group for cotton fabrics in mild dyeing system

Canxing Zhao [a,1], Rui Shi [a], Shouchun Li [a,b], Penghui Li [a], Xiaoxue Zhang [a], Guolin Tong [a,*]

a Jiangsu Co-Innovation Center of Efficient Processing and Utilization of Forest Resources, Nanjing Forestry University, Nanjing, 210018, PR China

b Sino-Italian Research Institute of Nanjing for Chemical Technology Development, Nanjing, 210037, PR China

***Corresponding author: Guolin Tong, gtong@njfu.edu.cn, 86-25-8542-8689.**

Contents

1. The ^{1}H NMR, ^{13}C NMR, ^{19}F NMR and IR spectra of the synthesized intermediate:

The carbon atom were replaced by a series of numbers from 1 to n (n = the number of carbon atom which are chemical shift with non-equivalent) and the non-equivalent hydrogen atoms were marked as lowercase alphabetic characters a, b, c, etc.

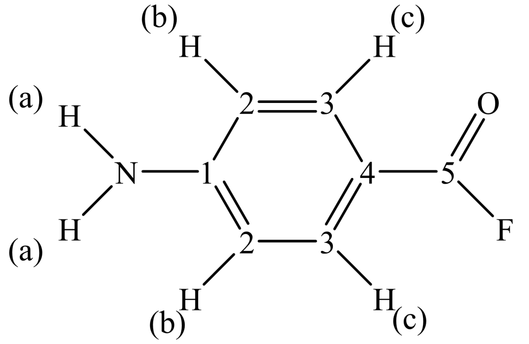

Figure S1. Chemical structure the dye intermediate.

^{1}H-NMR(600 MHz, CD_3OD, ppm)

a: 2.15

b: 7.65-7.68

c: 7.95-7.98

^{13}C-NMR (151 MHz, CD_3OD, ppm)

1: 144.60

2: 120.30

3: 132.02

4: 127.35

5: 172.20-169.89 J_{C-F}=349

^{19}F-NMR (565 MHz, CD_3OD, ppm): δ 17.52.

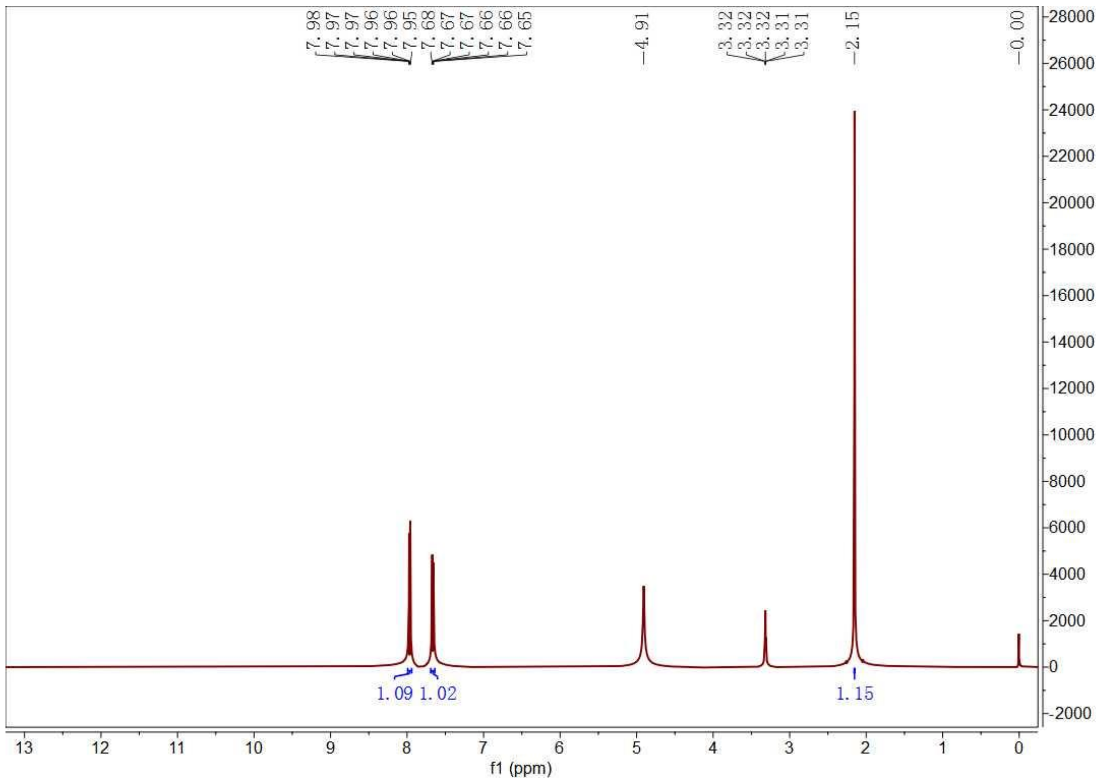

Figure S2. ^{1}H-NMR spectrum of the dye intermediate.

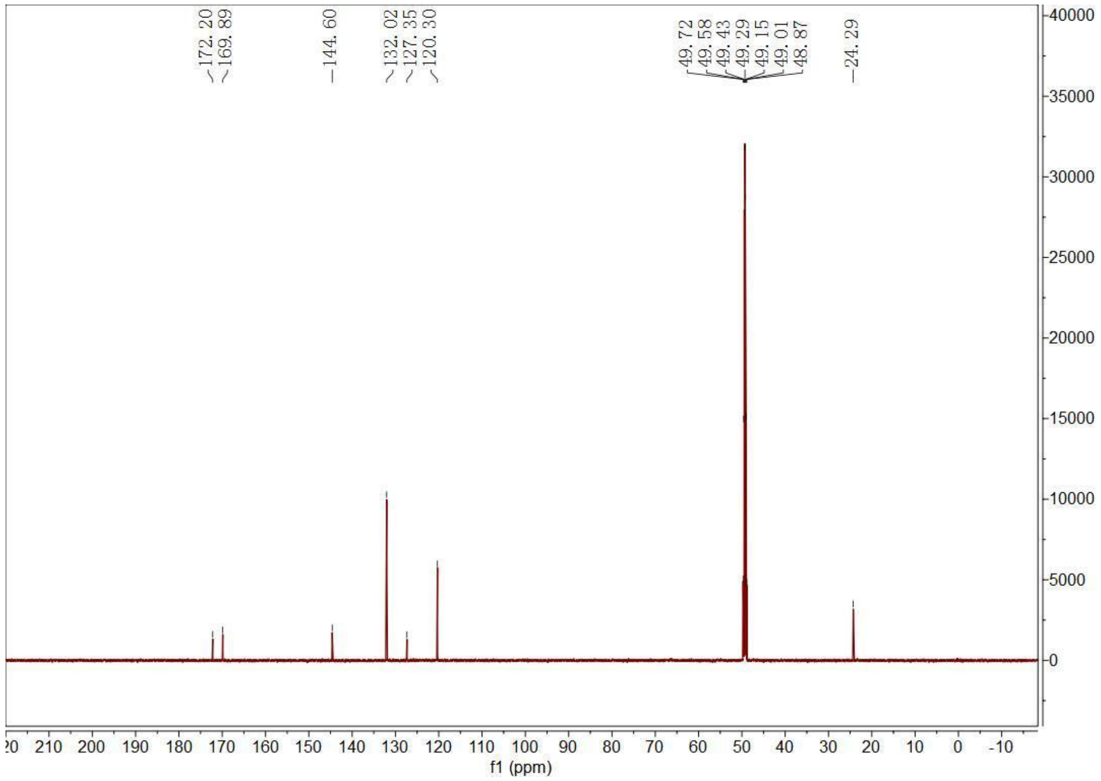

Figure S3. ^{13}C-NMR spectrum of the dye intermediate.

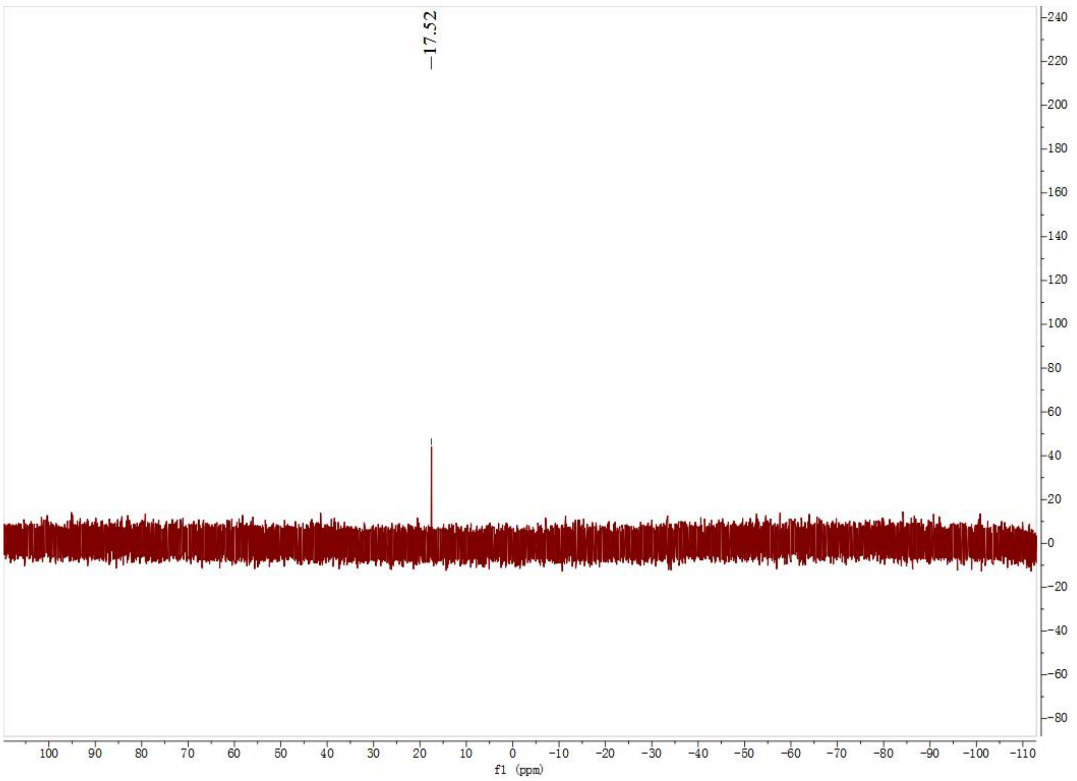

Figure S4. ^{19}F-NMR spectrum of the dye intermediate.

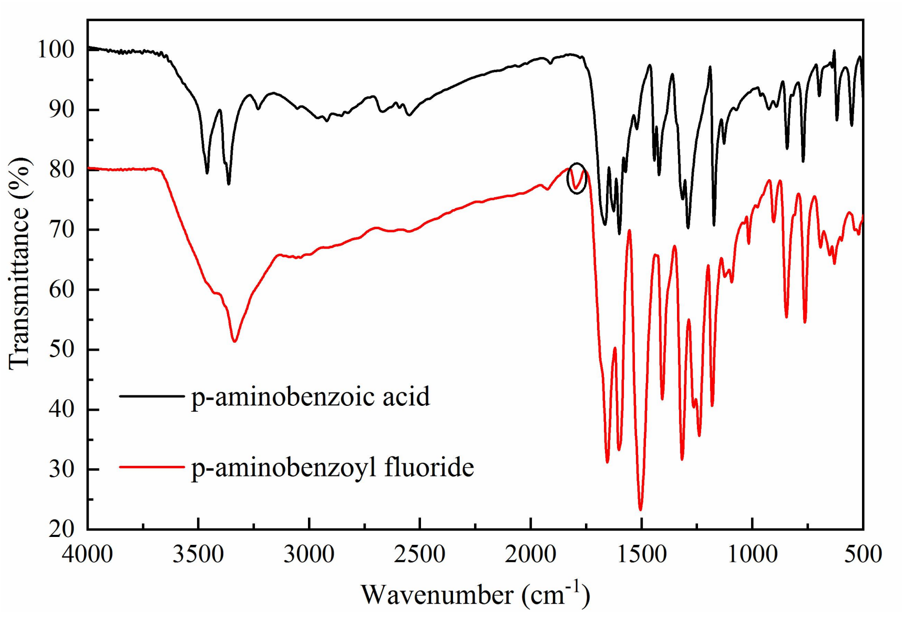

Figure S5. IR Spectra of the dye intermediate and 4-aminobenzoic acid.

2. The ^{1}H NMR, ^{13}C NMR, ^{19}F NMR spectra of the synthesized dyes

The carbon atom were replaced by a series of numbers from 1 to n (n = the number of carbon atom which are chemical shift with non-equivalent) and the non-equivalent hydrogen atoms were marked as lowercase alphabetic characters a, b, c, etc.

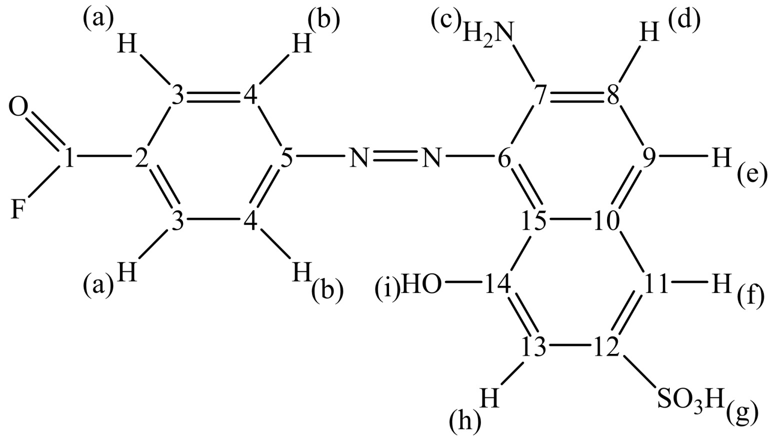

Figure S6. Chemical structure the Dye-1.

^{1}H-NMR(600 MHz, DMSO-d_6, ppm)

a: 8.00-7.98

b: 7.45-7.44

c: 7.25

d: 7.66

e: 8.07

f: 7.33

g: 7.56

h: 7.16

i: 7.94

^{13}C-NMR (151 MHz, DMSO-d_6, ppm)

1: 167.26-164.97 $J_{C\text{-}F}$=345.8

2: 129.93

3: 130.52

4: 115.41

5: 153.03

6: 122.25

7: 132.61

8: 120.95

9: 127.71

10: 124.88

11: 115.94

12: 131.42

13: 107.43

14: 146.69

15: 118.67

^{19}F-NMR (565 MHz, DMSO-d_6, ppm): δ 18.92.

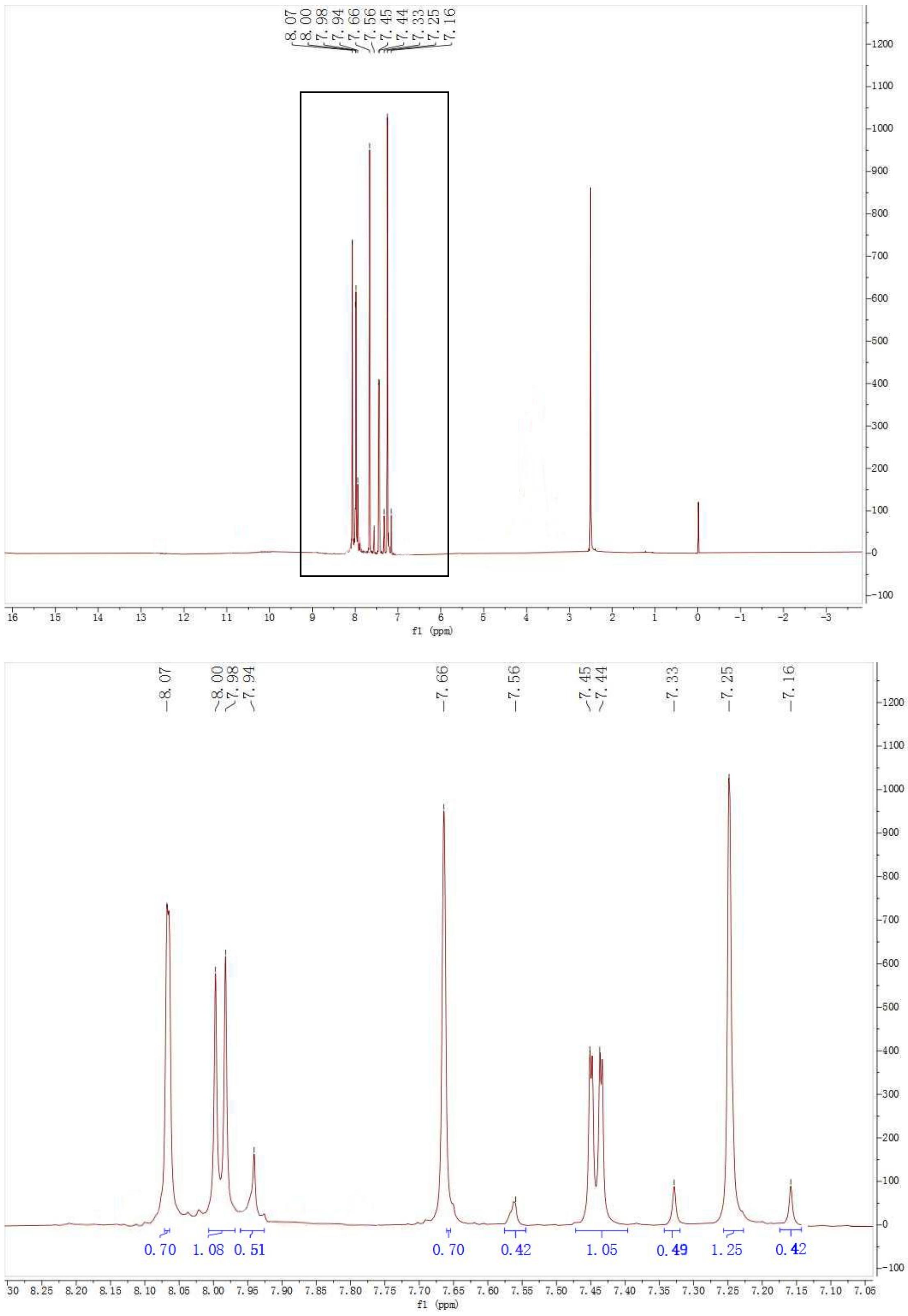

Figure S7. ^{1}H-NMR spectrum of the Dye-1.

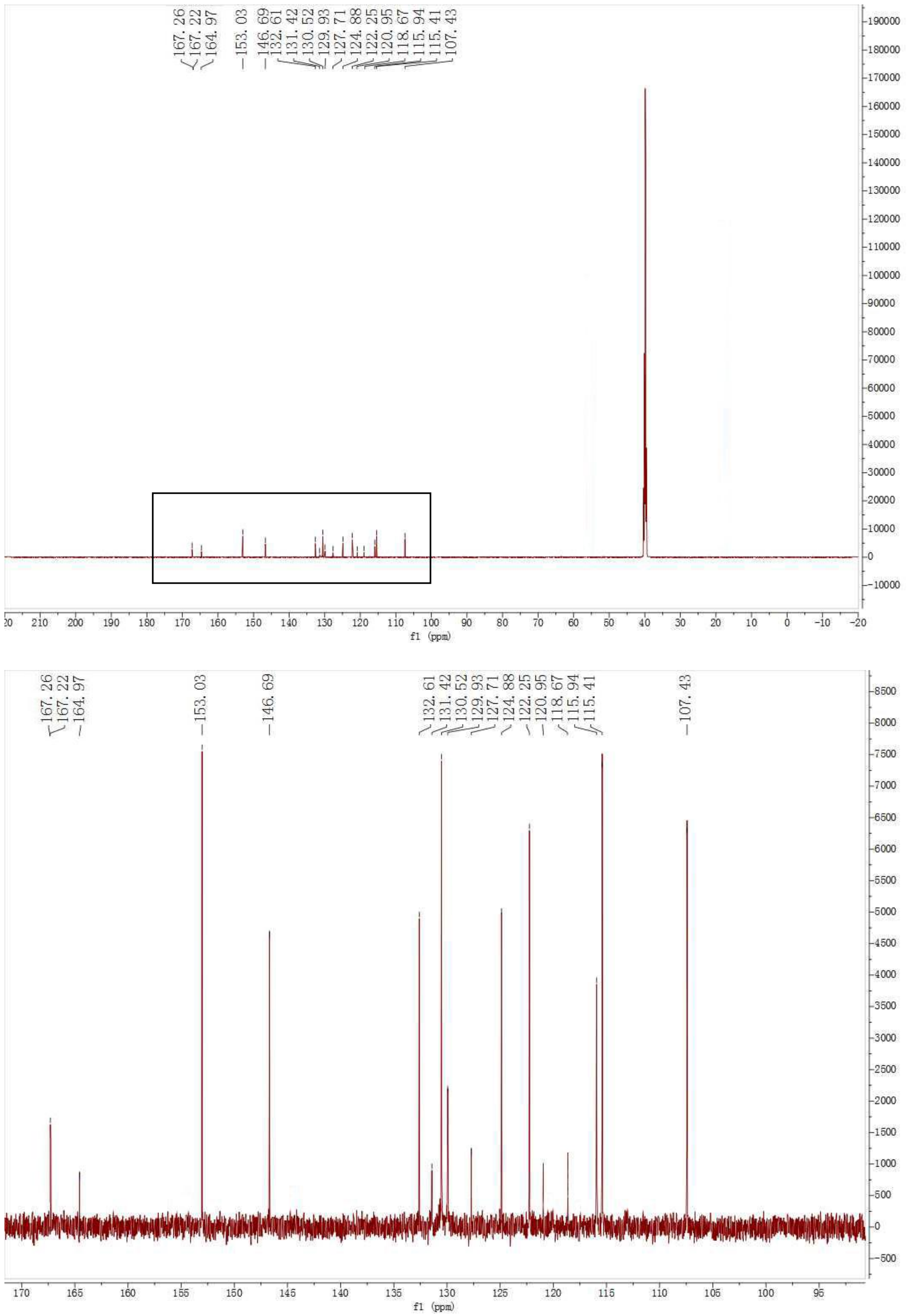

Figure S8. ^{13}C-NMR spectrum of the Dye-1.

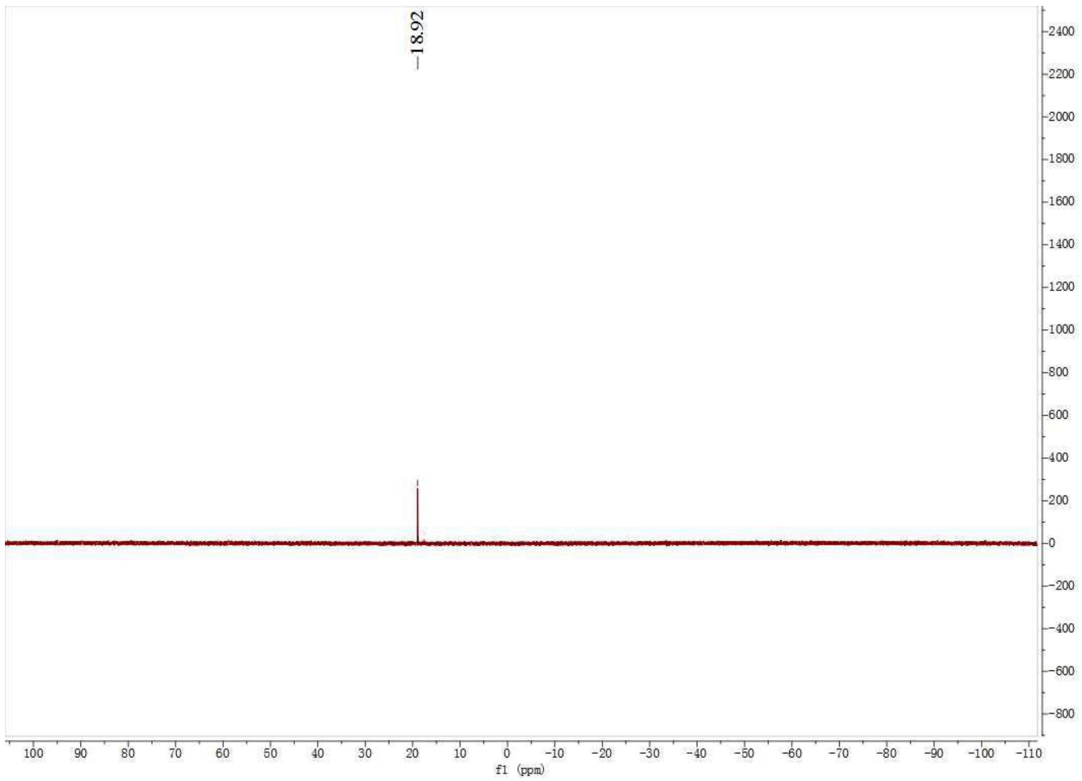

Figure S9. ^{19}F-NMR spectrum of the Dye-1.

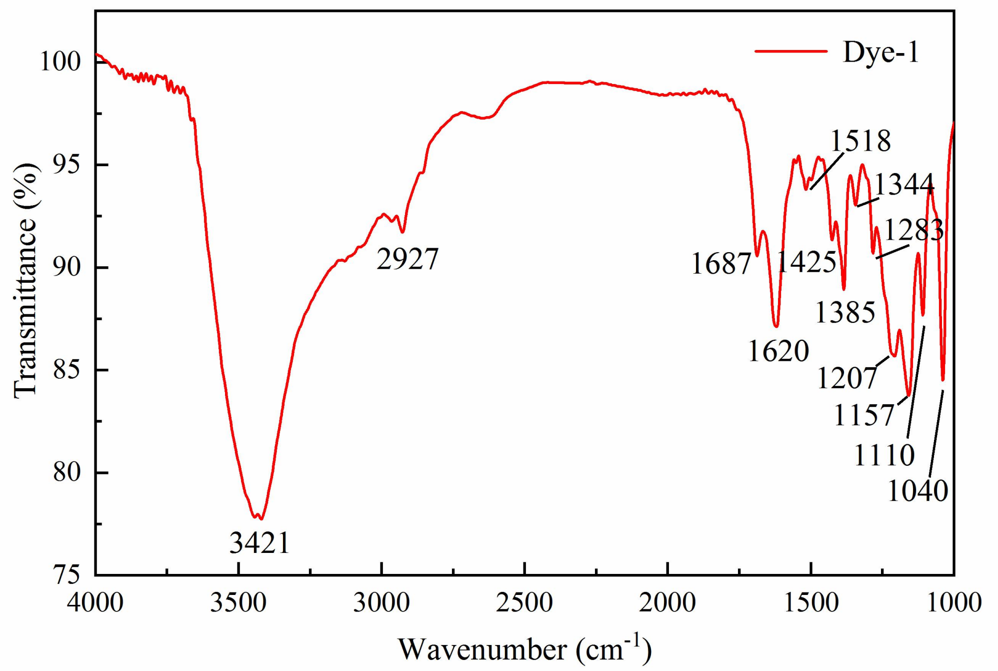

Figure S10. IR Spectrum of the Dye-1.

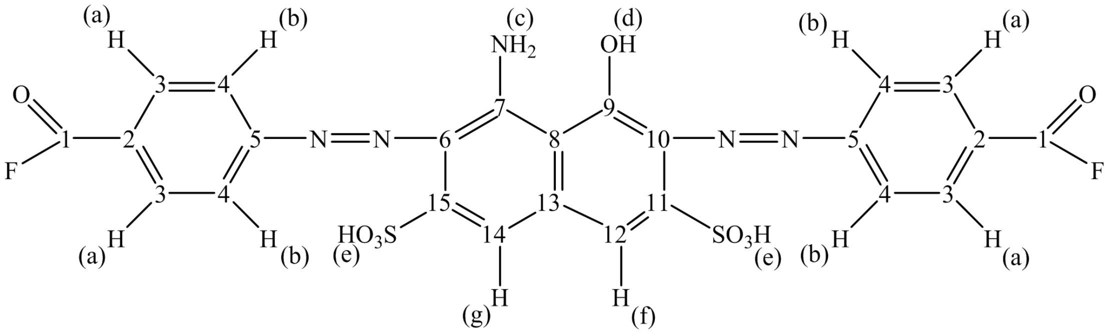

Figure S11. Chemical structure the Dye-2.

^{1}H-NMR(600 MHz, DMSO-d$_6$, ppm)

a: 8.00

b: 7.93

c: 6.64

d: 10.40

e: 8.26

f: 7.50

g: 7.45

^{13}C-NMR (151 MHz, DMSO-d$_6$, ppm)

1: 167.28-164.48 $J_{C\text{-}F}$=422.8

2: 130.66

3: 131.48

4: 120.97

5: 154.28

6: 130.41

7: 127.73

8: 115.60

9: 138.10

10: 125.09

11: 131.61

12: 118.53

13: 131.95

14: 117.46

15: 132.93

^{19}F-NMR (565 MHz, DMSO-d_6, ppm): δ 20.72.

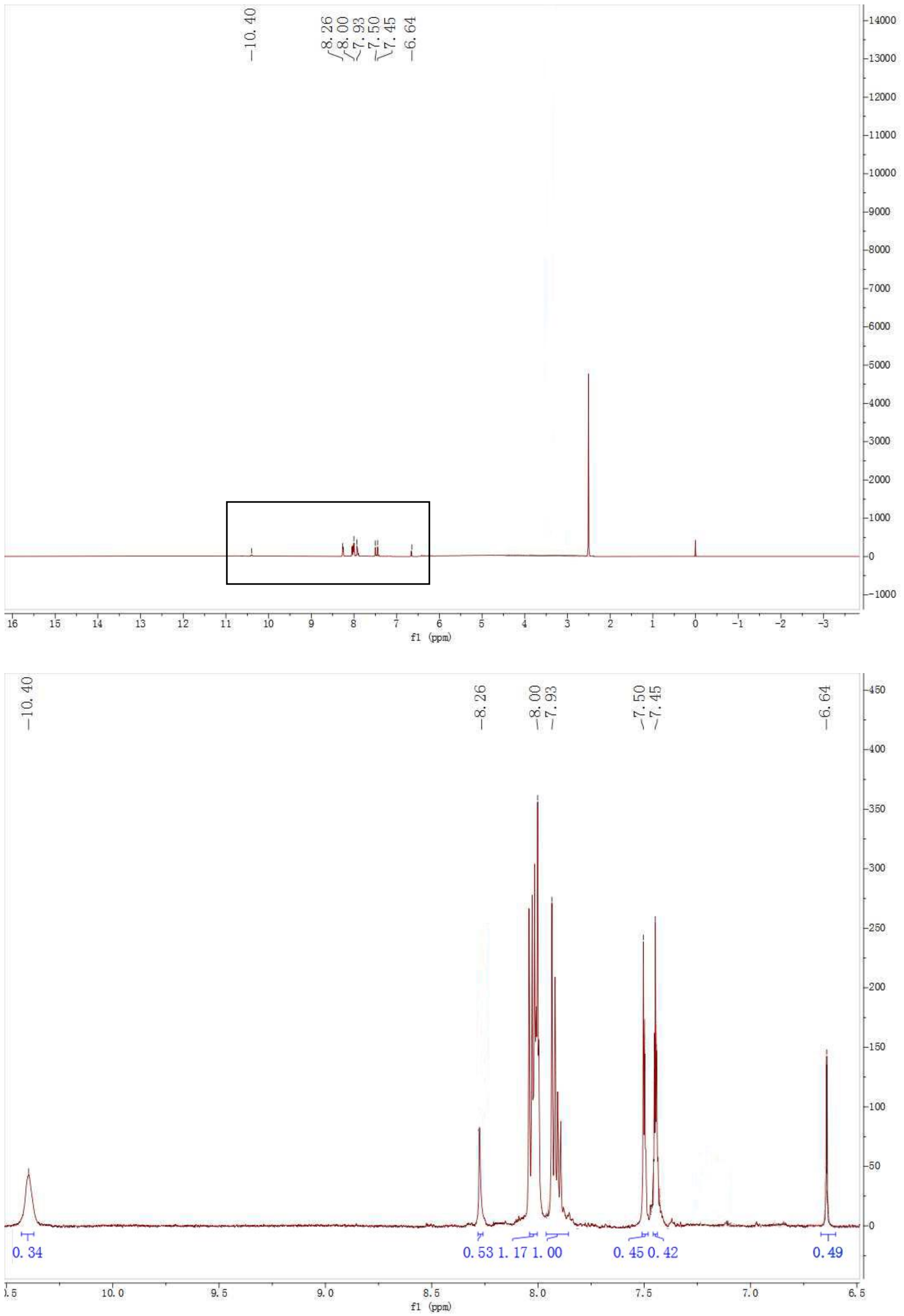

Figure S12. ^{1}H-NMR spectrum of the Dye-2.

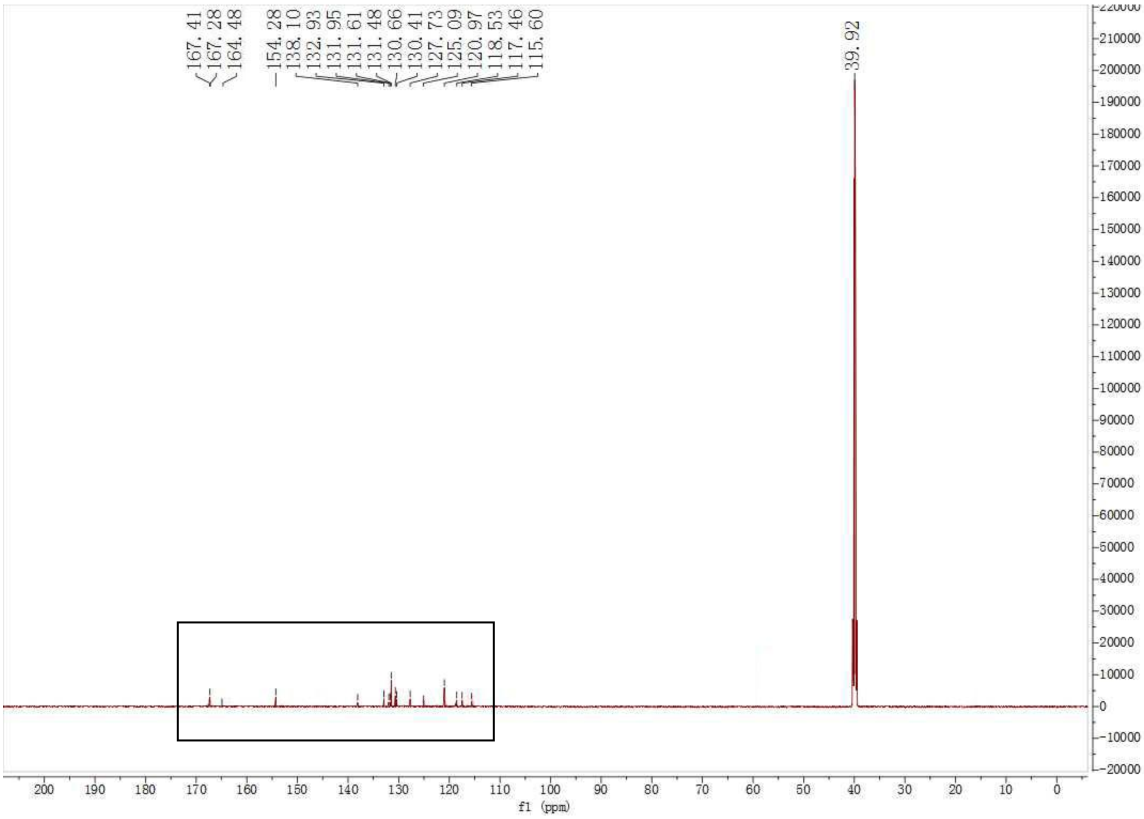

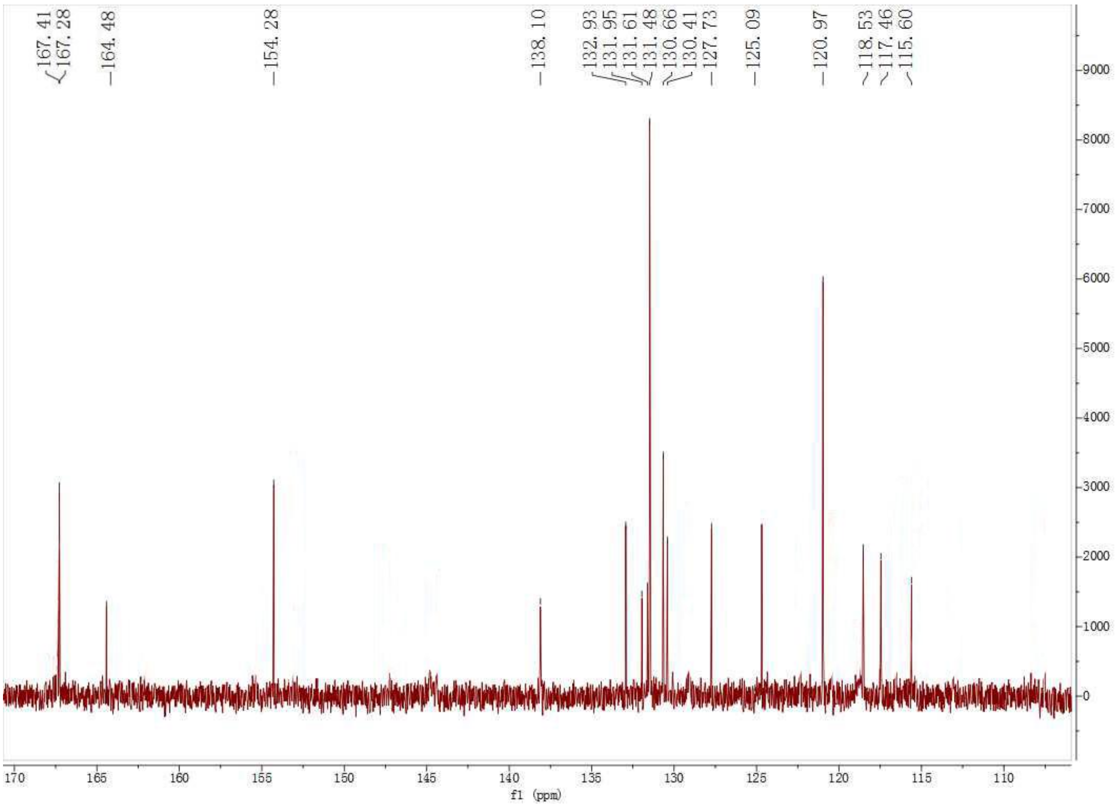

Figure S13. ^{13}C-NMR spectrum of the Dye-2.

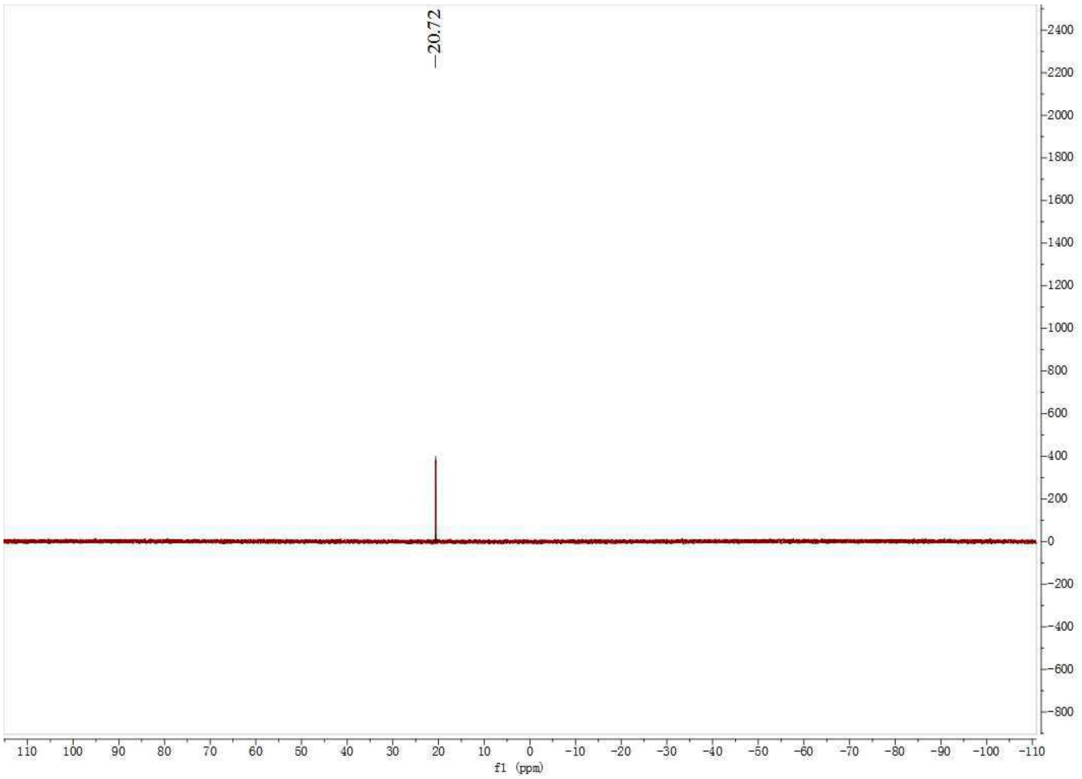

Figure S14. ^{19}F-NMR spectrum of the Dye-2.

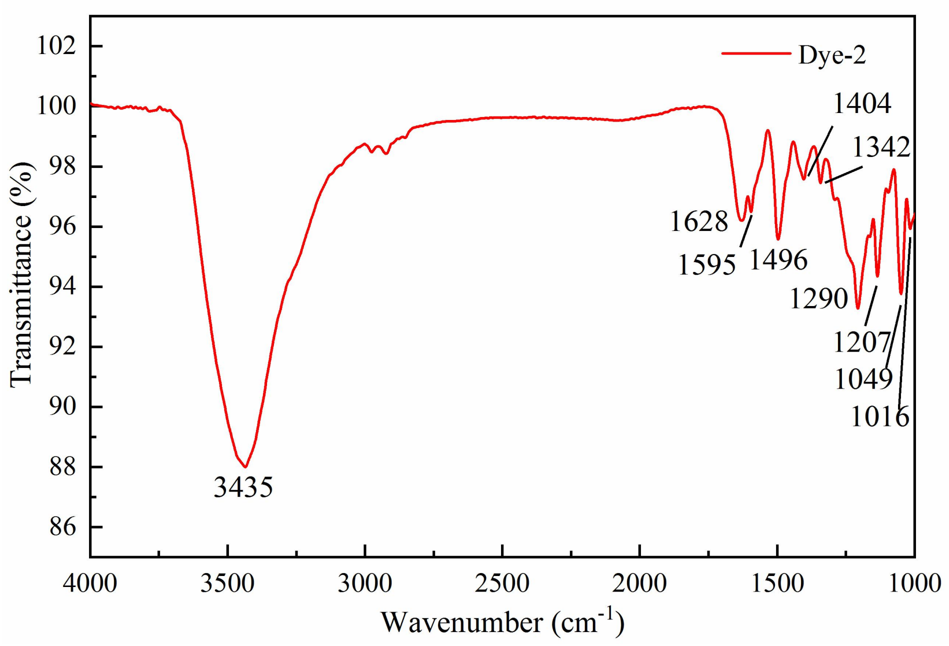

Figure S15. IR Spectrum of the Dye-2.
